# Strong tunable absorption enhancement in graphene using dielectric-metal core-shell resonators

**DOI:** 10.1038/s41598-017-00056-4

**Published:** 2017-02-17

**Authors:** Mingjie Wan, Yan Li, Jiawei Chen, Wenyang Wu, Zhuo Chen, Zhenlin Wang, Huitian Wang

**Affiliations:** 1 School of Physics and National Laboratory of Solid State Microstructures, Nanjing University, Nanjing, 210093 China; 2 Department of Mathematics and Physics, Zhengzhou Institute of Aeronautical Industry Management, Zhengzhou, 450015 China; 3 Collaborative Innovation Center of Advanced Microstructures, Nanjing, 210093 China

## Abstract

We theoretically investigate light absorption by a graphene monolayer that is coated on the outside of dielectric-metal core-shell resonators (DMCSRs). We demonstrate that light absorption of graphene can be greatly enhanced in such multi-layered core-shell architectures as a result of the excitation of the hybridized bonding plasmon resonance supported by the DMCSRs. We also demonstrate that the absorption enhancement in graphene can be easily tuned over a wide range from the visible to the near-infrared, and particularly the enhancement factor can be optimally maximized at any selective wavelength, by simultaneously varying the dielectric core size and the metal shell thickness. Our results suggest that the graphene-wrapped DMCSRs with strong and highly wavelength-tunable absorption enhancement in graphene could be attractive candidates for applications in graphene-based photodetectors and image sensors.

## Introduction

Graphene, a two-dimensional honeycomb lattice of sp^2^-hybridized carbon atoms, exhibits unique properties such as high intrinsic carrier mobility^1^, broad absorption spectrum of light^2^, high Young’s modulus^3^ and extraordinary thermal conductivity^4^, enabling it a powerful alternative for use in advanced optoelectronic devices^5^. Graphene is an excellent light-to-current converter with internal quantum efficiency close to 100%^6^, however, the relatively low absolute value of light absorption (~2.3%) for one atomic layer graphene^2, 7^ could severely limit its applications in many optoelectronic devices such as photodetectors^8^ and tunable modulators^9^. It has been demonstrated that graphene with appropriate doping could support either propagating^10–12^ or localized surface plasmon resonances^13–16^, and associated with the excitation of these graphene-plasmon resonances light absorption in graphene could be greatly enhanced. For example, Abajo *et al.* demonstrated that 100% light absorption could take place in a single patterned sheet of doped graphene^13^. However, this enhancement mechanism only works in the infrared region and is not applicable for the absorption enhancement in graphene in the visible and near-infrared (vis-NIR) spectral range, because of the absence of graphene-plasmon in the vis-NIR range^17^. In order to improve the interaction between graphene and vis-NIR light, many approaches have been employed. For example, the integration of graphene with an optical waveguide has been shown to allow the increase of the interaction length through coupling between the evanescent waves and graphene, and thus resulting in greatly enhanced graphene absorption^18^. Similarly, by placing graphene monolayer in optical micro-cavities such as resonant Fabry-Perot^19, 20^ and photonic crystal cavities^21, 22^, which enables light to pass through the graphene film multiple times, absorption enhancement in graphene has been demonstrated as a result of the increased effective interaction length. Recently, based on the ability of plasmonic nanostructures to concentrate light into subwavelength volumes and produce a dramatic enhancement of the local electric field^23^, the combination of graphene with plasmonic nanostructures has also been theoretically predicted and experimentally demonstrated as an efficient way to enhance the vis-NIR light absorption in graphene^24–33^. Although most of these metal-graphene hybrid nanostructures exhibit large absorption enhancement in graphene, the implementation of these nanostructures typically relies on high-cost and time-consuming top-down processes such as electron-beam lithography or focused-ion beam milling^24–27, 29–32^. Therefore, simple metal-graphene hybrid nanostructures that can greatly enhance graphene absorption and be readily prepared either chemically or physically are highly desirable.

Dielectric-metal core-shell resonators (DMCSRs), composed of a spherical dielectric core and a thin metal shell layer, are simple nanostructures that support both sphere and cavity plasmons^34^. Because of the finite thickness of the shell layer, these two primitive plasmons can interact with each other and result in hybridized bonding and antibonding modes^35^. The resonance wavelengths of these two hybridized plasmon modes can be broadly tuned by varying the relative dimensions of the core and shell^35^. Over the past few years, the DMCSRs have been routinely synthesized by using physical deposition process^36, 37^ or wet-chemistry approach^38^ where the metal shell can be as thin as ~2 nm^39^, and also have found widespread applications such as thermal-therapy^40^, low-threshold plasmonic lasing^41^, fluorescence emission shaping^42^, and refractive index sensor^43^. Motivated by these investigations, in this work, we propose the use of DMCSRs to enhance light absorption in graphene monolayer that is wrapped on the outside of the DMCSRs. We demonstrate that excitation of the hybridized bonding plasmon resonances supported by the DMCSRs can lead to a dramatic enhancement of the light absorption in the graphene layer, which can be easily tuned over a wide range from the visible to the near-infrared by varying either the dielectric core size or the thickness of the metal shell layer. Furthermore, we also demonstrate that there exists a particular combination of the core size and metal shell thickness to make the graphene absorption reach the maximum value at any selective wavelength.

## Results and Discussion

Throughout this paper, the scattering (absorption) of electromagnetic-plane waves from core-shell nanoparticles is analyzed using Mie theory^44^. In the calculations, the monolayer graphene is assumed to be 0.335-nm-thick^17^, and its permittivity within the vis-NIR region is described by a Drude-Lorentz model,

1 $${\varepsilon }_{gra}={\varepsilon }_{\infty }-\frac{{\omega }_{gra}^{2}}{{\omega }^{2}+i\omega {\gamma }_{gra}}+\sum _{j=1}^{m}\frac{{\rm{\Delta }}{\varepsilon }_{j}{{\rm{\Omega }}}_{j}^{2}}{{{\rm{\Omega }}}_{j}^{2}-{\omega }^{2}-i\omega {{\rm{\Gamma }}}_{j}},$$

where *m* = 3, *ε*_∞_ = 1.964, Δ*ε*_*j*_ = (6.99, 1.69, 1.53), *ħω*_*gra*_ = 6.02 eV, *ħγ*_*gra*_ = 4.52 eV, *ħΩ*_*j*_ = (3.14, 4.03, 4.59) eV, and *ħΓ*_*j*_ = (7.99, 2.01, 0.88) eV^45^. The metal is assumed to be silver, and its dielectric constant is described by a Drude model,

2 $${\varepsilon }_{Ag}=3.7-{\omega }_{Ag}^{2}/[\omega (\omega +i{\gamma }_{Ag})],$$

where *ω*_*Ag*_ = 1.38 × 10^16^ rad/s is the plasma frequency of bulk silver and *γ*_*Ag*_ is the frequency of electron collisions. The efficiencies of absorption in graphene (*Q*_*gra*_) and silver (*Q*_*Ag*_) components are given by *Q*_*gra*_ = *C*_*gra*_/*σ*_*geom*_ and *Q*_*Ag*_ = *C*_*Ag*_/*σ*_*geom*_, respectively, where *C*_*gra*_ and *C*_*Ag*_ are absorption cross section for the graphene and silver, respectively, and *σ*_*geom*_ = *πr* ^2^ is the geometrical cross-section with *r* being the outer radius of the core-shell nanoparticle. In order to reveal the essence of the underlying physics, we will begin our discussions by analyzing the ideal scenario where silver is assumed to be lossless (*γ*_*Ag*_ = 0 rad/s). In this case, only the graphene layer will absorb light (*Q*_*Ag*_ = 0). Later in this work, we will discuss the effects of metal loss on the graphene absorption.

**Figure 1:**
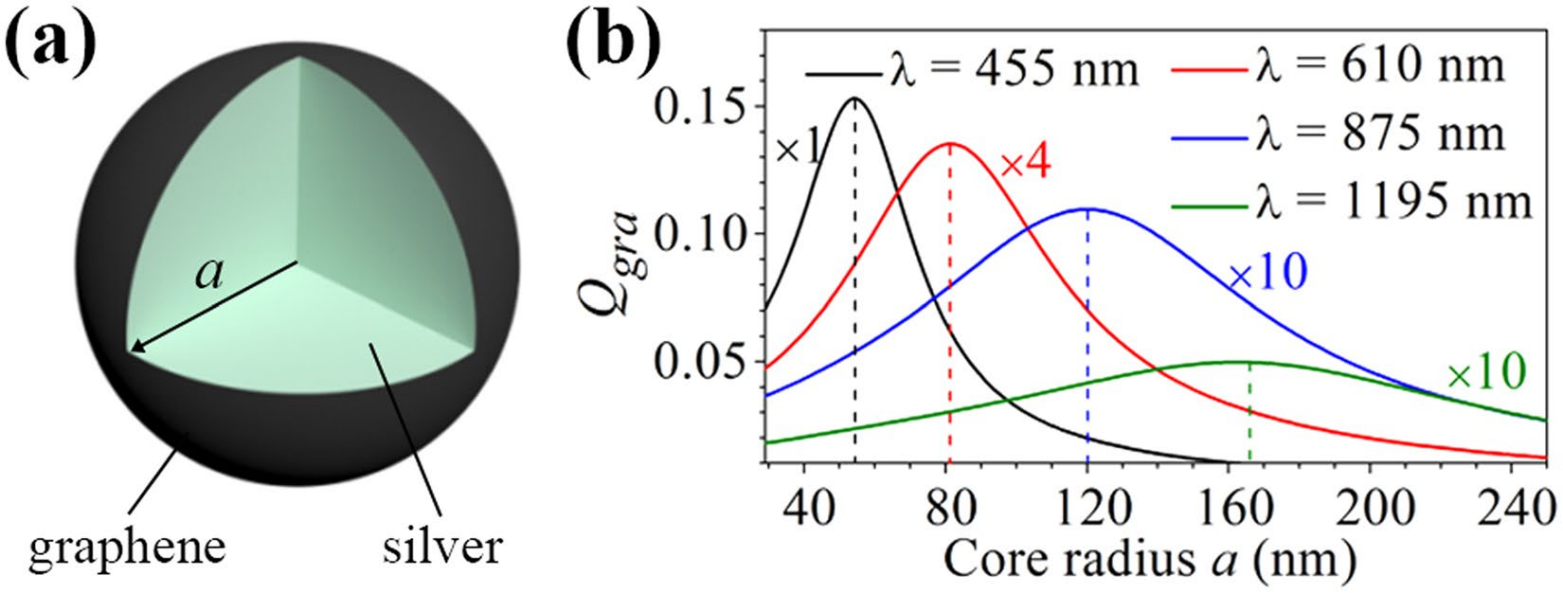
(**a**) Schematic of a graphene-wrapped solid silver nanosphere. (**b**) Graphene absorption efficiencies as a function of the radius of the silver nanosphere calculated at four different wavelengths of *λ* = 455 nm (black), *λ* = 610 nm (red), *λ* = 875 nm (blue) and *λ* = 1195 nm (green). For clarity, the graphene absorption efficiencies at the wavelengths of *λ* = 610 nm, *λ* = 875 nm and *λ* = 1195 nm are amplified 4, 10 and 10 times, respectively. For each wavelength, a dashed vertical line indicates a particular silver core radius with which the graphene absorption efficiency can achieve the maximum value.

Figure 1(a) schematically illustrates a composite nanoparticle consisting of a solid silver nanosphere wrapped directly by a monolayer graphene. For such a single-layered core-shell nanostructure, the only degree of freedom is the radius (*a*) of the silver nanosphere. Therefore, the achievable maximum graphene absorption efficiency at any given wavelength and the corresponding optimized core radius can be found by calculating the dependency of the absorption efficiency at this selective wavelength on the radius of the silver core. Figure 1(b) shows the efficiencies of absorption in graphene (*Q*_*gra*_) as a function of silver core radius calculated at four different vis-NIR wavelengths of *λ* = 455 nm, 610 nm, 875 nm, and 1195 nm. As indicated by dashed lines in Fig. 1(b), when the silver core radii are taken to be *a* = 54 nm, 81 nm, 120 nm, and 167 nm, the graphene absorption efficiency can achieve the maximum value at the given wavelengths of *λ* = 455 nm, 610 nm, 875 nm and 1195 nm, respectively, which reveal that the spectral position of the maximum graphene absorption can be tuned by varying the radius of the silver nanosphere. However, it should be noted that the achievable maximum absorption efficiency is very weak in the vis-NIR region and decrease with red-shifting the spectral position of graphene absorption by increasing the core radius [Fig. 1(b)]. For example, the achievable maximum absorption efficiency falls quickly from *Q*_*gra*_ ≈ 0.15 at *λ* = 455 nm to *Q*_*gra*_ ≈ 0.005 at *λ* = 1195 nm [Fig. 1(b)].

**Figure 2:**
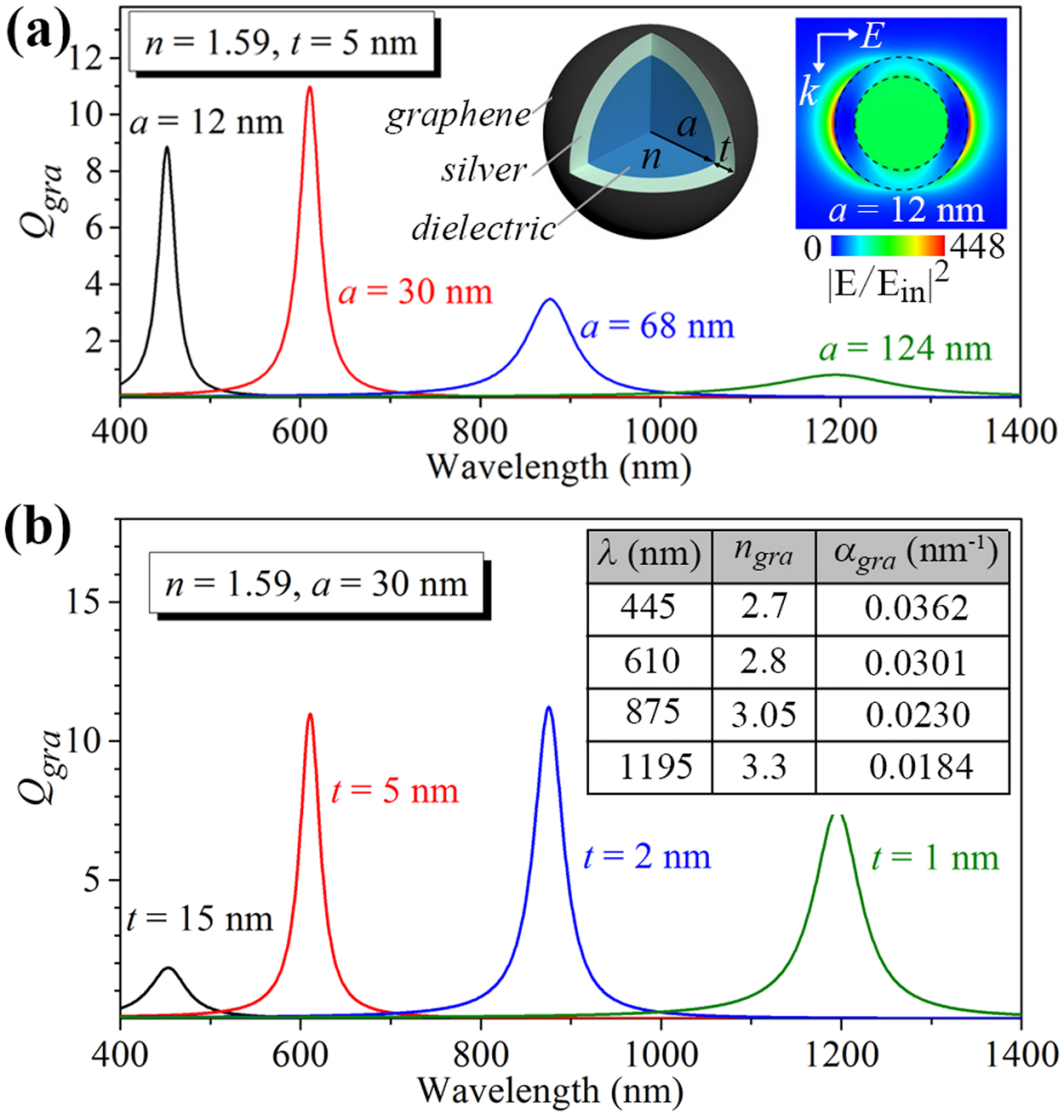
(**a**) Graphene absorption efficiency spectra for the graphene-wrapped DMCSRs with fixed dielectric core refractive index of *n* = 1.59 and silver shell thickness of *t* = 5 nm but different core radii. The left inset shows a schematic of a graphene-wrapped DMCSR. The right inset shows the normalized electric field intensity distribution at *λ* = 455 nm (corresponds to the core radius of *a* = 12 nm). (**b**) Graphene absorption efficiency spectra for the graphene-wrapped DMCSRs with fixed dielectric core refractive index of *n* = 1.59 and core radius of *a* = 30 nm but different silver shell thicknesses. The dielectric core radii in (**a**) and the silver shell thickness in (**b**) are chosen such that the absorption peaks appear at four given wavelengths of *λ* = 455 nm (black line), *λ* = 610 nm (red line), *λ* =875 nm (blue line) and *λ* = 1195 nm (green line). The inset table in (**b**) shows the refractive index and the absorption coefficient of monolayer graphene at the wavelengths of *λ* = 455 nm, 610 nm, 875 nm and 1195 nm.

In the following, we demonstrate that it is possible to greatly enhance the graphene absorption in the vis-NIR region by replacing the solid silver spheres in the above case [Fig. 1(a)] with the DMCSRs to form a graphene-wrapped DMCSR present in the left inset of Fig. 2(a). For such a two-layered core-shell nanostructure, there are three degrees of freedom including the radius (*a*) and refractive index (*n*) of the dielectric core, and the thickness (*t*) of the silver shell layer. Figure 2(a) shows the graphene absorption efficiency spectra calculated for the graphene-wrapped DMCSRs with different core radii while the refractive index of dielectric core and the thickness of the silver shell are fixed to *n* = 1.59 (e.g. polystyrene) and *t* = 5 nm. For a dielectric core radius of *a* = 12 nm, an absorption peak appears at the visible wavelength of *λ* = 455 nm and the graphene absorption efficiency at this wavelength reaches a value of *Q*_*gra*_ ≈ 8.8 [black line in Fig. 2(a)], which is about 59 times larger than that in a graphene-wrapped solid silver nanosphere [*Q*_*gra*_ ≈ 0.15, Fig. 1(b)]. The electric field intensity distribution is calculated at the absorption peak position (*λ* = 455 nm) for the core radius of *a* = 12 nm and shown in the right inset of Fig. 2(a), in which the electric fields with two lobes are found to be mostly localized on the outer surface of the graphene-wrapped DMCSR, revealing that the observed graphene absorption peak corresponds to the excitation of the hybridized dipolar bonding plasmon resonance. It has been demonstrated that the graphene-metal or bimetallic film based systems can exhibit higher field enhancement associated with the excitations of the propagating surface plasmon resonances in the Kretschmann or Otto configuration, and consequently improve the sensing performance of these film based systems^46–48^. Similarly, in our case, the intensity of the localized electric fields is found to be enhanced about 450 times of the incident field [right inset of Fig. 2(a)]. When the core radius is increased to *a* = 30 nm, 68 nm and 124 nm, the graphene absorption peak red-shifts to the wavelength of *λ* = 610 nm, 875 nm and 1195 nm, respectively [Fig. 2(a)]. Since the hybridized bonding plasmon resonance wavelengths are dependent on the relative dimensions of the core and shell^35^, there are multiple combinations of the core radius and the silver shell thickness with which the DMCSRs can resonate at the same wavelength. Indeed, the graphene absorption efficiency spectra calculated for the graphene-wrapped DMCSRs with the fixed core radius (*a* = 30 nm) and core refractive index (*n* = 1.59) but different silver shell thicknesses, presented in Fig. 2(b), show that the graphene absorption peak can also be tuned to the above four wavelengths by setting the silver shell thickness to *t* = 15 nm, 5 nm, 2 nm and 1 nm, respectively. It should be noted from the comparison between Fig. 2(a) and (b) that although the graphene absorption peak could be tuned to the same selective wavelength by varying either the dielectric core size (*a*) or the silver shell thickness (*t*), the obtained absorption efficiency could be quite different. For example, for a graphene-wrapped DMCSR with the combination of parameters (*n* = 1.59, *a* = 124 nm, *t* = 5 nm) the graphene absorption peak appears at the wavelength of *λ* = 1195 nm and its efficiency reaches the value of *Q*_*gra*_ ≈ 0.8 [green line in Fig. 2(a)]. When the combination of parameters is taken to (*n* = 1.59, *a* = 124 nm, *t* = 5 nm), the graphene absorption peak appears at the same wavelength of *λ* = 1195 nm, but its efficiency now can reach a much higher value of *Q*_*gra*_ ≈ 7.54 [green line in Fig. 2(b)].

**Figure 3:**
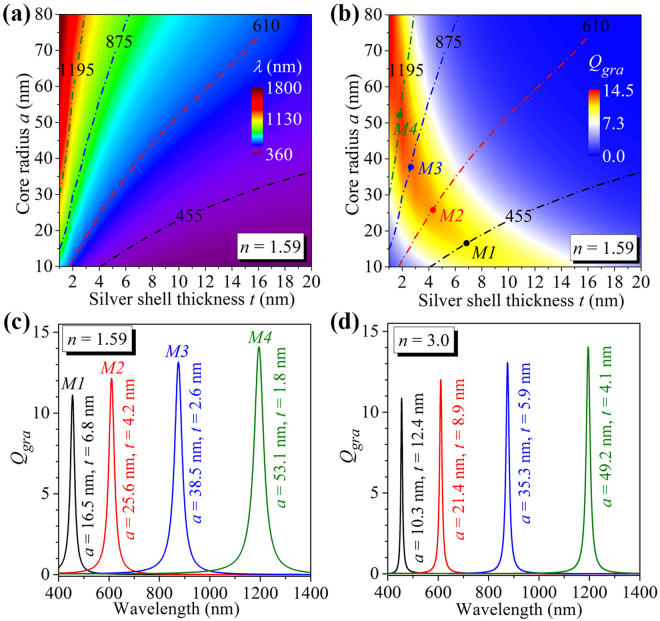
Two-dimensional colour map plot of spectral positions (**a**) and efficiencies (**b**) of the peaks that appear in the graphene absorption efficiency spectra calculated for the graphene-wrapped DMCSRs with the same core refractive index of *n* = 1.59 but different core sizes and silver shell thicknesses. Dashed lines in (**a**) and (**b**) outline the 455 nm (black), 610 nm (red), 875 nm (blue), and 1195 nm contours (green). Marked points M1, M2, M3 and M4 in (**b**) indicate the particular combinations of the silver shell thickness and the core radius where the graphene absorption efficiency can achieve the maximum value at the wavelengths of 455 nm, 610 nm, 875 nm and 1195 nm, respectively. (**c**) Graphene absorption efficiency spectra for the graphene-wrapped DMCSRs with fixed core refractive index of n = 1.59 and the optimized parameters corresponding to the marked points M1, M2, M3, and M4 in (**b**). (**d**) Graphene absorption efficiency spectra for the graphene-wrapped DMCSRs with fixed core refractive index of *n* = 3.0, in which the structural parameters are optimized so that the graphene absorption efficiencies can achieve the maximum values at the wavelengths of 455 nm, 610 nm, 875 nm and 1195 nm.

In order to locate the maximum graphene absorption efficiency at a given wavelength for graphene-wrapped DMCSRs with a fixed dielectric core refractive index (*n* = 1.59), the graphene absorption efficiency spectra are calculated with the simultaneous variations of the dielectric core radius (10 nm ≤ *a *≤ 80 nm) and the silver shell thickness (1 nm ≤ *t *≤ 20 nm). The spectral position of the graphene absorption peak and the corresponding efficiency are collected from all these absorption efficiency spectra and plotted in Fig. 3(a) and (b), respectively, as functions of the core radius and the silver shell thickness, which actually demonstrate an absorption optimization process. A contour of any given wavelength is first determined in Fig. 3(a) by directly connecting points (combinations of the core radius and the silver shell thickness) where the absorption peak appears at the same particular wavelength. As shown in Fig. 3(a), four dashed lines are used to outline the 455 nm, 610 nm, 875 nm, and 1195 nm contours. The obtained contours are then superimposed to the two-dimensional (2D) colour map plot of efficiencies at the absorption peaks [dashed lines in Fig. 3(b)], along which the variation of the graphene absorption efficiency at a particular wavelength can be clearly seen. As marked by the points M1, M2, M3, and M4 in Fig. 3(b), the graphene absorption efficiencies at these points can achieve the maximum value at the wavelengths of *λ* = 455 nm, 610 nm, 875 nm, and 1195 nm, respectively. Figure 3(c) shows the absorption efficiency spectra calculated for the graphene-wrapped DMCSRs with four particular sets of parameters corresponding to the marked points M1 (*a* = 16.5 nm, *t* = 6.8 nm), M2 (*a* = 25.6 nm, *t* = 4.2 nm), M3 (*a* = 38.5 nm, *t* = 2.6 nm), and M4 (*a* = 53.1 nm, *t* = 1.8 nm) in Fig. 3(b). As expected, the absorption peaks with the efficiencies as high as *Q*_*gra*_ ≈ 11.1, 12.1, 13.0, and 14.1 can be observed at the wavelengths of *λ* = 455 nm, 610 nm, 875 nm and 1195 nm, respectively, which are generally 2–3 orders of magnitude larger than that in graphene-wrapped solid silver nanospheres [*Q*_*gra*_ ≈ 0.15 at *λ* = 455 nm, *Q*_*gra*_ ≈ 0.034 at *λ* = 610 nm, *Q*_*gra*_ ≈ 0.011 at *λ* = 875 nm, and *Q*_*gra*_ ≈ 0.005 at *λ* = 1195 nm, Fig. 1(b)]. The above demonstrated optimization process can also be applied to the graphene-wrapped DMCSRs with a dielectric core having higher refractive index and give rise to a set of optimized parameters for any given wavelength. Figure 3(d) shows the absorption efficiency spectra calculated for the graphene-wrapped DMCSRs with the fixed core refractive index of *n* = 3.0 (e.g. AlGaAs or silicon oxynitride) and the parameters (*a* = 10.3 nm, *t* = 12.4 nm), (*a* = 21.4 nm, *t* = 8.9 nm), (*a* = 35.3 nm, *t* = 5.9 nm), and (*a* = 49.2 nm, *t* = 4.1 nm), which are optimized for the wavelengths of *λ* = 455 nm, 610 nm, 875 nm, and 1195 nm, respectively. By comparing Fig. 3(d) and (c), it is found that for the graphene-wrapped DMCSRs with different core refractive indexes the achievable maximum absorption efficiencies at a given wavelength are almost same. In both cases, the achieved maximum absorption efficiencies at the longer wavelength require a combination of larger dielectric core size and thinner silver shell thickness [Fig. 3(c) and (d)]. Furthermore, the comparison between Fig. 3(d) and (c) also reveals that for a given wavelength the optimized core radius and the silver shell thickness in the graphene-wrapped DMCSRs with a relatively small core refractive index are respectively larger and smaller than that in the graphene-wrapped DMCSRs with a relatively large core refractive index. For example, for the graphene-wrapped DMCSRs with a core refractive index of *n* = 1.59, the maximum absorption efficiency at the wavelength of *λ* = 1195 nm occurs when the core radius and the silver shell thickness are taken to *a* = 53.1 nm and *t* = 1.8 nm [green line in Fig. 3(c)]. By using a dielectric core with a higher refractive index of *n* = 3.0, the optimized core radius and silver shell thickness such that the absorption efficiency at *λ* = 1195 nm can reach the maximum value are respectively found to be decreased to *a* = 49.2 nm and increased to *t* = 4.1 nm [green line in Fig. 3(d)].

**Figure 4:**
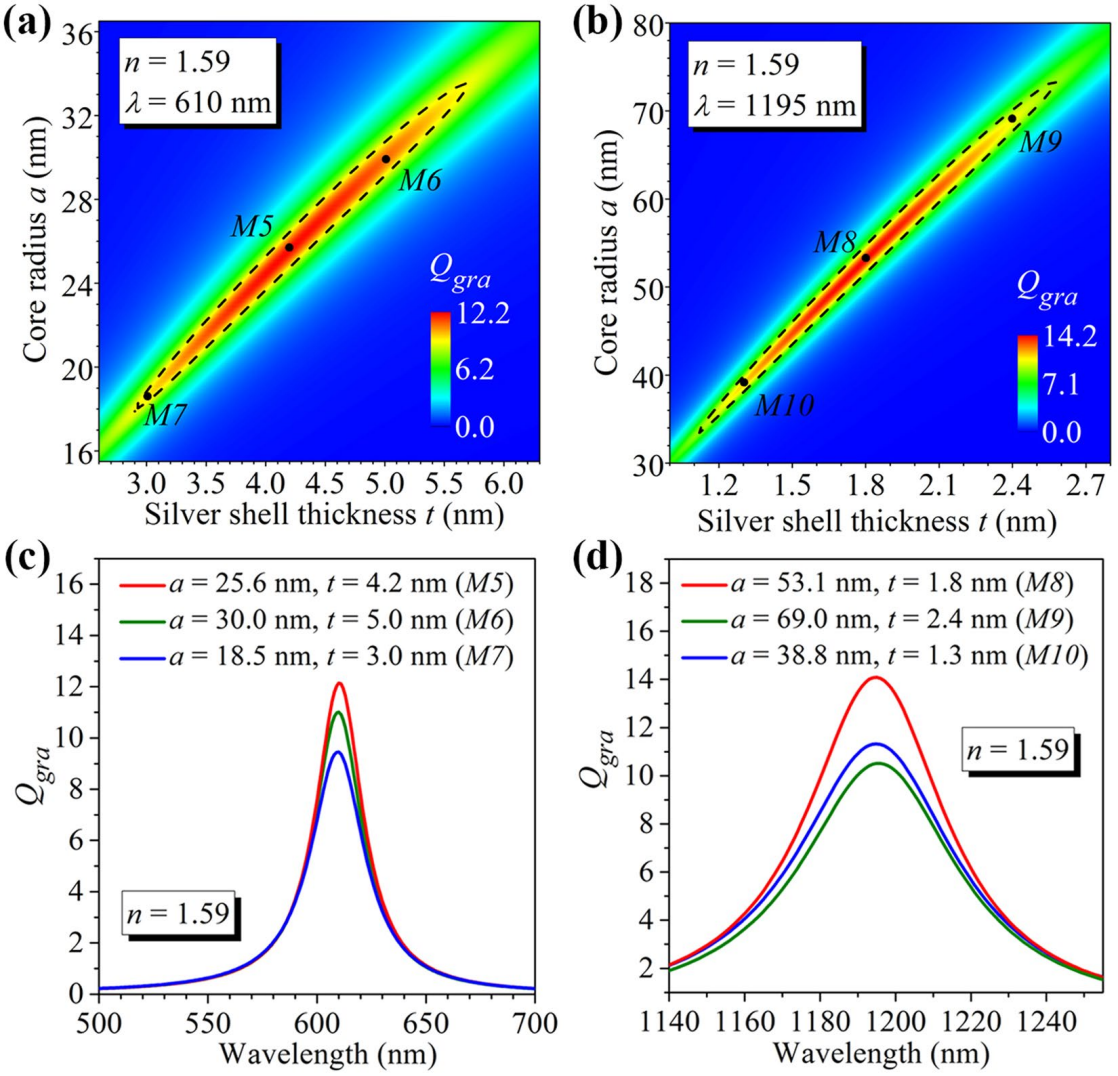
Graphene absorption efficiency as a function of core radius and silver shell thickness calculated at the given wavelength of *λ* = 610 nm (**a**) and the given wavelength of *λ* = 1195 nm (**b**) for the graphene-wrapped DMCSRs with fixed core refractive index of *n* = 1.59. Black dashed lines in (**a**) and (**b**) outline the boundary where the absorption efficiency is equal to 9. Marked points M5, M6, and M7 in (**a**) and M8, M9, and M10 in (**b**) locate inside the outlined regions. (**c**) Absorption efficiency spectra of the graphene-wrapped DMCSRs with parameters corresponding to the marked points M5, M6, and M7 in (**a**). (**d**) Absorption efficiency spectra of the graphene-wrapped DMCSRs with parameters corresponding to the marked points M8, M9, and M10 in (**b**).

Although it has been demonstrated that the graphene absorption efficiency at a given vis-NIR wavelength can reach its maximum value only for the graphene-wrapped DMCSRs with a particular set of optimized parameters, the graphene-wrapped DMCSRs with other parameters can still exhibit relatively large absorption efficiency. To demonstrate this, the graphene absorption efficiencies are calculated at the wavelengths of *λ* = 610 nm and *λ* = 1195 nm for the graphene-wrapped DMCSRs with a fixed core refractive index of *n* = 1.59 and plotted in Fig. 4(a) and (b), respectively, as functions of the silver shell thickness and core radius. Dashed lines are used in both Fig. 4(a) and (b) to outline the boundary where the graphene absorption efficiency is equal to *Q*_*gra*_ = 9, which is about 265 times larger than the achievable maximum absorption efficiency of *Q*_*gra*_ ≈ 0.034 at *λ* = 610 nm and about 1800 times larger than the achievable maximum absorption efficiency of *Q*_*gra*_ ≈ 0.005 at *λ* = 1195 nm in graphene-wrapped solid silver nanospheres [Fig. 1(b)]. It is seen from Fig. 4(a) that relatively large graphene absorption efficiency (*Q*_*gra*_ ≥ 9) at the wavelength of *λ* = 610 nm can be obtained within the core radius range of 18 nm ≤ *a *≤ 33 nm and the silver shell thickness range of 2.9 nm ≤ *t *≤ 5.7 nm. For the wavelength of *λ* = 1195 nm, large graphene absorption efficiency (*Q*_*gra*_ ≥ 9) can be obtained within the core radius range of 31 nm ≤ *a *≤ 72 nm and the silver shell thickness range of 1.1 nm ≤ *t *≤ 2.6 nm [Fig. 4(b)]. The graphene absorption efficiency spectra of the graphene-wrapped DMCSRs with the parameters of (*a* = 25.6 nm, *t* = 4.2 nm), (*a* = 30 nm, *t* = 5 nm), and (*a* = 18.5 nm, *t* = 3 nm) corresponding to the points *M5*, *M6* and *M7* located inside the outlined region of Fig. 4(a), and the parameters of (*a* = 53.1 nm, *t* = 1.8 nm), (*a* = 69 nm, *t* = 2.4 nm), and (*a* = 38.3 nm, *t* = 1.3 nm) corresponding to the marked points *M8*, *M9* and *M10* of Fig. 4(b), are calculated and shown in Fig. 4(c) and (d). As expected, absorption peaks in Fig. 4(c) and (d) appear around the wavelength of *λ* = 610 nm and *λ* = 1195 nm, respectively, which reveals that a flexible choice of the parameters of the graphene-wrapped DMCSRs is allowed for the achievement of a relatively large hybridized dipolar bonding plasmon resonance induced absorption enhancement in graphene.

**Figure 5:**
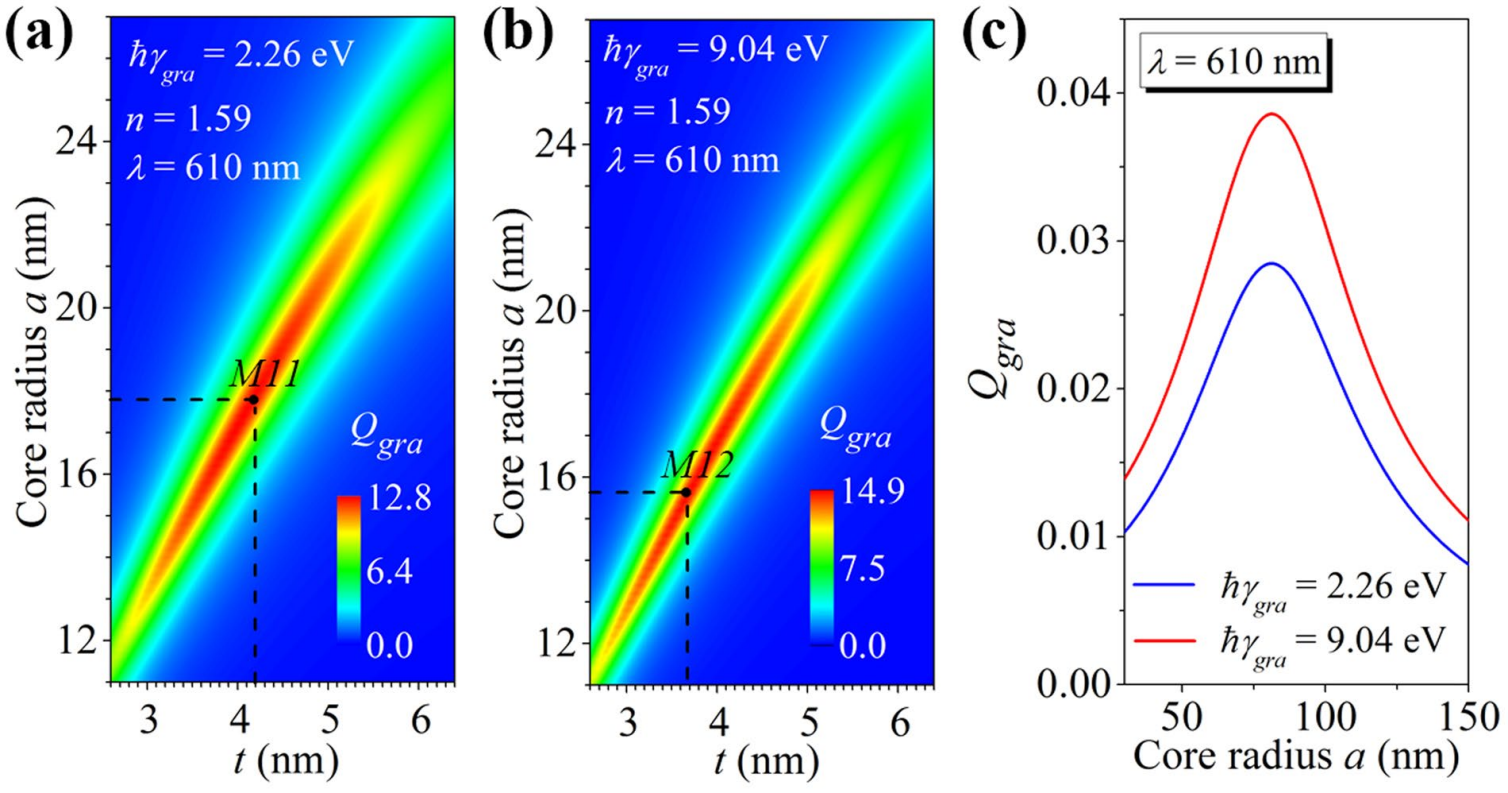
Graphene absorption efficiency as a function of core radius and silver shell thickness calculated at the given wavelength of *λ* = 610 nm for the graphene-wrapped DMCSRs with the fixed core refractive index of *n* = 1.59 and the graphene electron collision frequency being *ħγ*_*gra*_ = 2.26 eV (**a**) and *ħγ*_*gra*_ = 9.04 eV (**b**). Marked points M11 in (**a**) and M12 in (**b**) indicate the particular combinations of the silver shell thickness and the core radius where the graphene absorption efficiency can achieve the maximum value at the wavelength of 610 nm. (**c**) Graphene absorption efficiencies as a function of the radius of the silver nanosphere calculated at the wavelengths of *λ* = 610 nm and different graphene electric collision frequencies of *ħγ*_*gra*_ = 2.26 eV (blue line) and *ħγ*_*gra*_ = 9.04 eV (red line).

It is worth noting that in the proposed graphene-wrapped structures the graphene has a spherical shape, which may change the dielectric function of graphene. In order to examine the effect of the possible changes of the dielectric function on the graphene absorption enhancement, the graphene electron collision frequency (*γ*_*gra*_) of the Drude-Lorentz model is varied in the calculations. Figure 5(a) and (b) show the graphene absorption efficiencies of the graphene-wrapped DMCSRs with a fixed core refractive index of *n* = 1.59 calculated at the wavelength of *λ* = 610 nm for *ħγ*_*gra*_ = 2.26 eV and 9.04 eV, respectively. As marked by the points *M11* (*a* = 18 nm, *t* = 4.2 nm) in Fig. 5(a) and *M12* (*a* = 15.6 nm, *t* = 3.7 nm) in Fig. 5(b), the graphene absorption efficiencies at these two points can achieve the maximum value of *Q*_*gra*_ ≈ 12.8 and 14.9, respectively. These two values are clearly on the same level as the achievable maximum absorption efficiency of *Q*_*gra*_ ≈ 12.1 obtained in the graphene-wrapped DMCSRs at *λ* = 610 nm for *ħγ*_*gra*_ = 4.52 eV [Fig. 3(c)]. For comparison, the graphene absorption efficiencies of the graphene-wrapped solid silver spheres are also calculated at the wavelength of *λ* = 610 nm for two different graphene electron collision frequencies of *ħγ*_*gra*_ = 2.26 eV and 9.04 eV, and plotted as a function of silver core radius in Fig. 5(c). It is seen that when the silver core radius is taken to be *a* = 81 nm, the graphene absorption efficiency at *λ* = 610 nm for *ħγ*_*gra*_ = 2.26 eV and 9.04 eV can achieve the maximum value of *Q*_*gra*_ ≈ 0.028 [blue line in Fig. 5(c)] and 0.039 [red line in Fig. 5(c)], respectively, which are closed to the achievable maximum absorption efficiency of *Q*_*gra*_ ≈ 0.034 obtained in the graphene-wrapped solid silver spheres at *λ* = 610 nm for *ħγ*_*gra*_ = 4.52 eV [Fig. 1(b)]. Therefore, even when *ħγ*_*gra*_ is varied from 2.26 eV to 9.04 eV, the achievable maximum absorption efficiency of the graphene-wrapped DMCSRs can be much larger than that of the graphene-wrapped solid silver nanospheres, and thus indicating that the proposed graphenen absorption enhancement mechanism is quite robust against the changes in the dielectric function of graphene.

**Figure 6:**
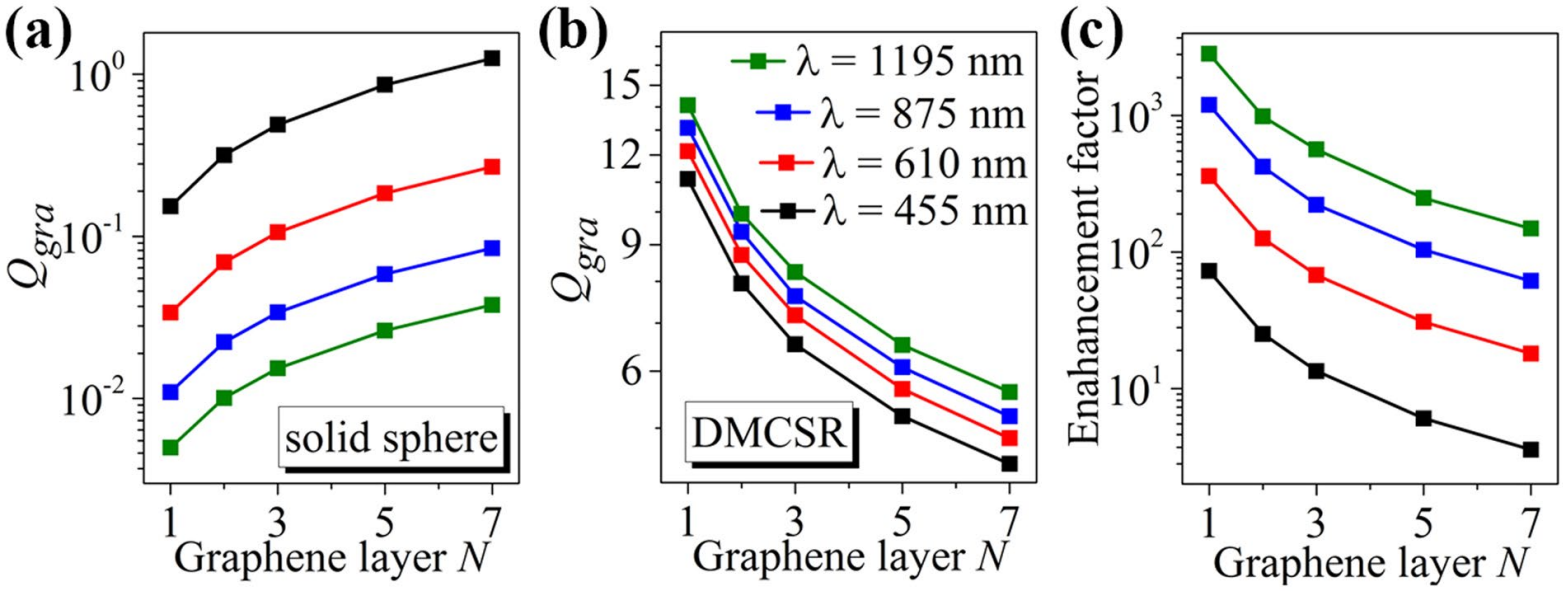
Maximum achievable graphene absorption efficiencies for the solid silver nanospheres (**a**) and the DMCSRs (**b**) wrapped by graphene with different layers at four different wavelengths of *λ* = 455 nm, 610 nm, 875 nm, and 1195 nm. (**c**) Enhancement factors of the graphene absorption efficiencies at the wavelengths of *λ* = 455 nm, 610 nm, 875 nm, and 1195 nm.

In the above cases, only single-layer graphene is coated on the outside of the solid silver spheres and DMCSRs. Now, the multilayer graphene with the layer number of *N* and the thickness of 0.335 × *N* nm is considered. By using the optimization process demonstrated in Fig. 1(b), the maximum achievable graphene absorption efficiencies can be obtained for the solid silver nanospheres wrapped by graphene with different layers at any given wavelength. Figure 6(a) summarizes these maximum absorption efficiencies at four different vis-NIR wavelengths of *λ* = 455 nm, 610 nm, 875 nm, and 1195 nm as a function of graphene layer. It is seen from Fig. 6(a) that at all of the given wavelengths the maximum achievable graphene absorption efficiencies increase with increasing the layer of the graphene. For example, when the graphene layer is increased from *N* = 1 to *N* = 5, the achievable maximum absorption efficiency increases from *Q*_*gra*_ ≈ 0.15 to 0.86 at *λ* = 455 nm [black line with symbols, Fig. 6(a)]. Similarly, by using the optimization process demonstrated in Fig. 3(a) and (b), the maximum achievable graphene absorption efficiencies at the wavelengths of *λ* = 455 nm, 610 nm, 875 nm, and 1195 nm for the graphene-wrapped DMCSRs with different graphene layers are also extracted and plotted in Fig. 6(b) as a function of the graphene layer, in which the refractive index of the dielectric core is fixed to *n* = 1.59. In this case, the maximum achievable graphene absorption efficiencies are found to decrease with increasing the layer of the graphene. For example, when the graphene layer is increased from *N* = 1 to *N* = 5, the achievable maximum absorption efficiency decreases from *Q*_*gra*_ ≈ 14.1 to 6.5 at *λ* = 1195 nm [green line with symbols, Fig. 6(b)]. Figure 6(c) summarizes the enhancement factor defined as the ratio of the maximum achievable graphene absorption efficiency in graphene-wrapped DMCSRs to that in graphene-wrapped solid silver spheres with the same graphene layer at the same wavelength. It is seen from Fig. 6(c) that for the same graphene layer the enhancement factor is larger at the longer wavelength. Furthermore, Fig. 6(c) shows that at any given wavelength the graphene absorption enhancement is decreased by about one order of magnitude when the graphene layer increases from *N* = 1 to *N* = 7, which suggests that the proposed DMCSRs are more suitable for enhancing light absorption of single-layer graphene.

**Figure 7:**
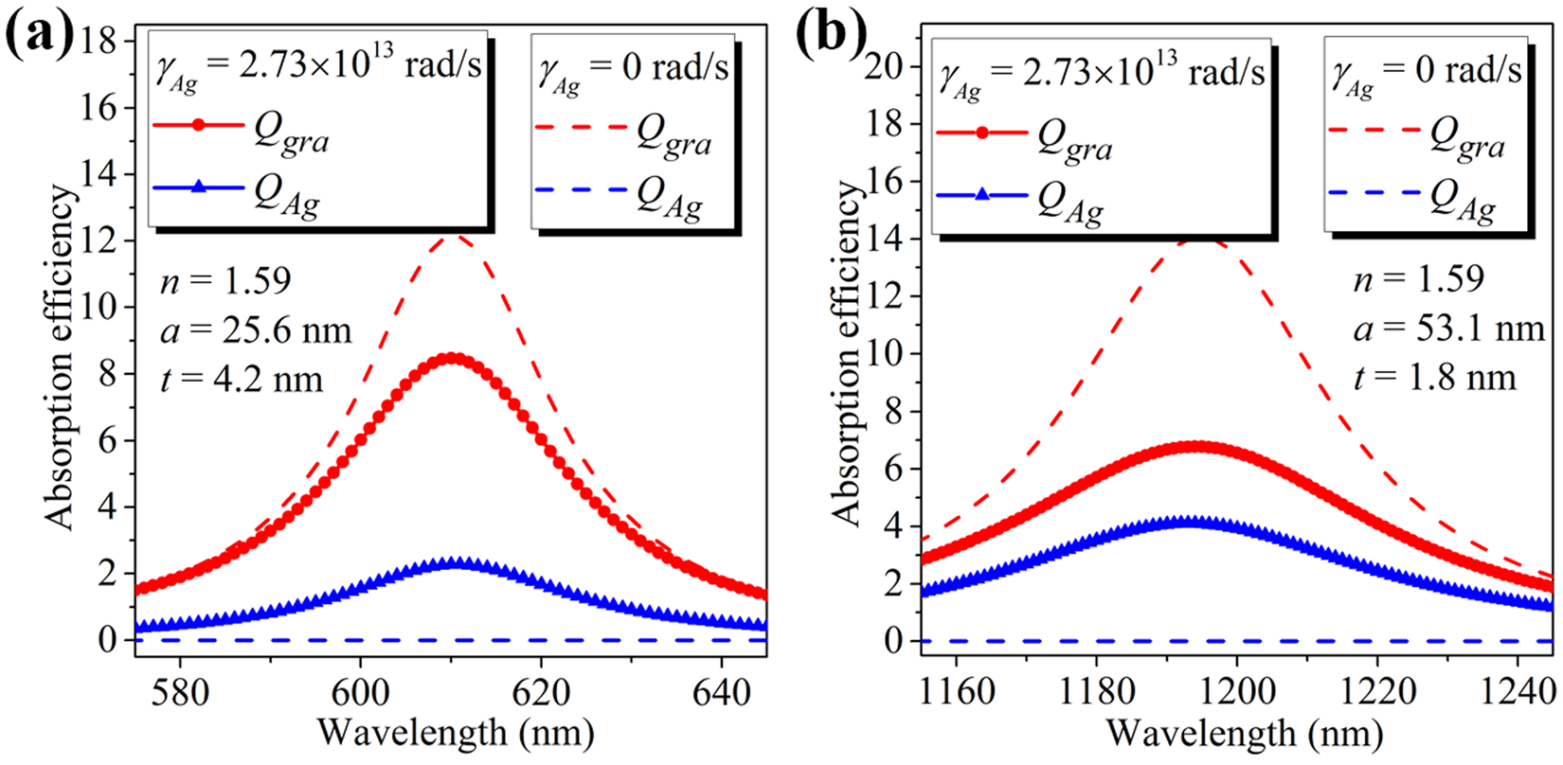
Graphene (red curves) and silver (blue curves) absorption efficiency spectra calculated for the graphene-wrapped DMCSRs with fixed core refractive index of *n* = 1.59 and the permittivity of silver being described by the lossless Drude model (*γ*_*Ag*_ = 0 rad/s, dashed lines) and the lossy Drude model (*γ*_*Ag*_ = 2.73 × 10^13^ rad/s, solid symbols), in which the parameters are optimized so that the graphene absorption efficiency can reach its maximum value at the wavelength of *λ* = 610 nm (**a**) and the wavelength of *λ* = 1195 nm (**b**).

In the practice the metal loss has to be taken into account. Figure 7(a) shows both the graphene (*Q*_*gra*_) and silver (*Q*_*Ag*_) absorption efficiency spectra calculated for the graphene-wrapped DMCSRs with fixed core refractive index of *n* = 1.59, in which the permittivity of silver is taken from the lossy Drude model with the frequency of electron collisions being *γ*_*Ag*_ = 2.73 × 10^13^ rad/s, and the combination of the core radius and the silver shell thickness is optimized so that the graphene absorption efficiency can reach its maximum value at the wavelength of *λ* = 610 nm. By using the above demonstrated optimization process, it can be found that the optimized core radius and silver shell thickness in the presence of metal loss are *a* = 25.6 nm and *t* = 4.2 nm, which are the same as those in the graphene-wrapped DMCSRs without metal loss [marked point *M2* in Fig. 3(b)]. For direct comparison, Fig. 7(a) also presents the graphene absorption efficiency spectrum of the MDGCSR with a lossless silver core and the optimized parameters. It is clearly seen from Fig. 7(a) that when *γ*_*Ag*_ = 2.73 × 10^13^ rad/s is taken into account in the Drude model the achievable maximum graphene absorption efficiency drops from *Q*_*gra*_ ≈ 12.1 [red dashed line] to *Q*_*gra*_ ≈ 8.5 [red solid circles], while the silver absorption efficiency correspondingly increases from *Q*_*Ag*_ = 0 [blue dashed line] to *Q*_*Ag*_ ≈ 2.3 [blue solid triangles]. In addition, Fig. 7(b) shows the graphene and silver absorption efficiency spectra with and without the metal loss calculated for the graphene-wrapped DMCSRs with fixed core refractive index of *n* = 1.59 and parameters of (*a* = 25.6 nm, *t* = 4.2 nm), which are optimized for the wavelength of *λ* = 1195 nm. Compared with the results for the graphene-wrapped DMCSR with a lossless silver shell [dashed lines in Fig. 7(b)], it is again found that the silver absorption efficiency is increased to *Q*_*Ag*_ ≈ 4.1 and the achievable maximum graphene absorption efficiency in the presence of metal loss decreases from *Q*_*gra*_ ≈ 14.1 to *Q*_*gra*_ ≈ 6.8. Although the finite dissipation could lead to the reduction in the achievable maximum graphene absorption efficiency, the graphene-wrapped DMCSR with a lossy silver shell could still be optimized to achieve a relatively large graphene absorption efficiency, for example, *Q*_*gra*_ ≈ 6.8 at the wavelength of *λ* = 1195 nm, which is about 1360 times larger than the achievable maximum graphene absorption efficiency at *λ* = 1195 nm in a graphene-wrapped solid silver nanosphere [*Q*_*gra*_ ≈ 0.005, Fig. 1(b)]. In addition, it is seen from Fig. 7 that the silver shell thickness needs to be decreased to achieve the maximum absorption in graphene at the longer wavelength. For DMCSRs with thinner silver shell, the cavity and sphere plasmon modes can interact with each other more strongly^38^, which could lead to the increased electric field intensity within the silver shell. Therefore, at the longer wavelength the silver absorption efficiency becomes larger. Consequently, the electric fields distributed outside the silver shell are less enhanced, resulting in a relatively small enhancement of the light absorption in the graphene layer at the longer wavelength. For this reason, the absorption efficiency ratio of the graphene to the silver is found to be relatively large (*Q*_*gra*_/*Q*_*Ag*_ ≈ 3.7) at the short wavelength of *λ* = 610 nm [Fig. 7(a)] and relatively small (*Q*_*gra*_/*Q*_*Ag*_ ≈ 1.7) at the long wavelength of *λ* = 1195 nm [Fig. 7(b)].

In summary, we demonstrate that the graphene-wrapped dielectric-metal core-shell resonators exhibit large graphene absorption enhancement arising from the excitation of hybridized bonding plasmon resonance supported by the DMCSRs. We show that the core-shell aspect ratio dependence of the hybridized plasmon resonance allows the spectral position of the graphene absorption enhancement to be easily tuned over a wide range from the visible to the near-infrared by varying the relative dimensions of the dielectric core and silver shell. We also demonstrate that the graphene absorption enhancement can be maximized at any given vis-NIR wavelength by simultaneously varying the dielectric core size and the silver shell thickness. Although the graphene absorption can reach the maximum at a given vis-NIR wavelength only for the graphene-wrapped DMCSRs with a particular set of optimized parameters, a flexible choice of the parameters of the DMCSRs is allowed for the achievement of relatively large graphene absorption. We suggest that the proposed graphene-wrapped DMCSRs can be prepared by using physical deposition process or wet-chemistry method to coat dielectric spheres with a metal shell layer^36–39^, followed by electrostatic wrapping of graphene sheets on the surfaces of the as-prepared DMCSRs^49^. The strong optical absorption in graphene and excellent tunability make the proposed graphene-wrapped DMCSRs attractive candidates for optoelectronic applications. We also hope our strategy could be straightforwardly applied to enhance absorption in other quasi-2D materials.

## Methods

The plane wave scattering (absorption) by a spherical concentric core-shell nanoparticle is solved analytically using full-wave Mie theory^44^, with the software Matlab. Since the considered spherical nanoparticles are much smaller than the wavelength of the interest, only the dipolar effects in Mie theory is taken into account. Initially, the silver is assumed to be perfect metal without loss. In this case, we can either integrate the dissipation power over the volume of the graphene shell to directly obtain the graphene absorption efficiency (*Q*_*gra*_), or calculate the graphene absorption efficiency based on the energy conservation: *Q*_*gra*_ = *Q*_*abs*_ = *Q*_*ext*_ – *Q*_*sca*_ (because only the graphene can absorb light). In the final part of the paper, we considered the effect of realistic metal loss on the graphene absorption. Since both graphene and silver can absorb light, the only way to calculate the losses caused by the silver and the graphene shells is to integrate the dissipation power over the volume of the silver shell and the graphene shell, respectively.
